# Periodontal disease and neuroinflammation in multiple sclerosis: a systematic review of current evidence

**DOI:** 10.3389/fdmed.2026.1701357

**Published:** 2026-02-20

**Authors:** Srijanani Santhanakrishnan, Karunanidhi Kannappan, Chandrasekaran Krithika, Chitathoor Sridhar, Jaideep Mahendra

**Affiliations:** 1 Department of Research, Meenakshi Academy of Higher Education and Research, Chennai, India; 2 Oral Medicine and Radiology, Meenakshi Ammal Dental College, Meenakshi Academy of Higher Education and Research, Chennai, India; 3 Internal Medicine, Meenakshi Academy of Higher Education and Research, Chennai, India; 4 Department of Periodontics, Meenakshi Ammal Dental College and Hospital, Meenakshi Academy of Higher Education and Research, Chennai, India

**Keywords:** autoimmune response, neurodegeneration, oral microbiome, oral-Immune axis, periodontal disease

## Abstract

**Background:**

Multiple Sclerosis (MS) is a chronic, immune-mediated neurological disorder characterized by demyelination and neurodegeneration. Emerging evidence suggests a link between MS and Periodontal Diseases (PD) through shared immune-inflammatory pathways. This review assesses the association between periodontal diseases and multiple sclerosis, focusing on immune-inflammatory interactions and clinical correlations. Despite emerging evidence, the strength of association remains unclear due to methodological heterogeneity.

**Aim:**

To review and evaluate the literature on the epidemiological association between PD and MS in adults.

**Materials and methods:**

A systematic search was conducted in PubMed, Scopus, and Cochrane. Studies with full text articles that are available in English, without time restrictions, that assessed periodontitis, oral microbiome, and salivary biomarkers in relation to MS were included. Observational studies evaluating clinical, microbiological, or immunological associations were selected. Data extraction covered periodontal parameters, salivary biomarkers, periodontal pathogens and disease severity. The risk of bias was evaluated using Newcastle-Ottawa Scale.

**Results:**

The findings indicated that patients with MS had poorer periodontal health, when compared to healthy controls. Dysbiosis in the oral microbiome was observed, with a higher abundance of periodontal pathogens. Patients with MS exhibited elevated neutrophil-lymphocyte ratios and total oxidative stress, indicating a potential link between systemic inflammation and periodontal dysbiosis. While some studies established positive association between PD and MS, others highlighted the need for further investigation due to inconsistent findings in periodontal parameters between MS patients and controls.

**Conclusion:**

Despite methodological heterogeneity, the available limited evidence indicates the association between periodontitis and MS. This highlights the need for standardized periodontal assessments in research involving MS and suggests that periodontal care may hold potential as an adjunct in management of MS.

## 1 Introduction

Multiple sclerosis (MS) is an auto-immune, inflammatory and neurodegenerative disorder described by axon loss of central nervous system and immune-mediated demyelination. In young adults, MS is considered to be one of the most prevalent causes of neurologic disturbances. The global prevalence of MS in 2020 was 35.9 per 100,000 people (1). Although several systemic factors such as Epstein–Barr virus, vitamin D deficiency, and smoking are recognized, the role of oral health—particularly periodontal disease—has received limited systematic evaluation. The etiology of MS remains largely unknown; however, it is widely recognized as a multifactorial disease influenced by genetic susceptibility and environmental triggers. Genetic predisposition has a significant impact, recent research showing links between MS and particular alleles of human leukocyte antigen (HLA) complex, mainly HLA-DRB1. Environmental factors such as infections caused by the Epstein–Barr virus, deficiency in vitamin D, smoking, and gut microbiome alterations have been implicated in disease pathogenesis. These factors collectively contribute to immune dysregulation, leading to chronic neuroinflammation and demyelination within the central nervous system (2).

Gingivitis and Periodontitis are the most common inflammatory diseases of periodontium (3). Gingivitis is a mild form of reversible gum disease characterised by gingival inflammation and gingival bleeding. On the other hand, periodontitis is an irreversible disease of periodontium characterized by inflammation and damage of supporting tooth structures including gingiva, cementum, bone, periodontal ligaments and etc., Periodontitis is triggered by presence of dental biofilm in oral cavity and lead to exacerbation of the disease (3). Even though the infection is localized to the periodontium, their significant effects on systemic health have been advocated in literature (4, 5).

Periodontal Disease (PD) is found to be linked with increased levels of inflammatory cytokines such as interleukin-6 (IL-6), interleukin-1beta (IL-1β), and tumor necrosis factor-alpha (TNF-α). By modifying expression tight junction proteins like occludin and claudin, these mediators could compromise Blood-Brain Barrier (BBB) and enhance permeability (4). Furthermore, virulence factors and lipopolysaccharides (LPS) released by periodontal pathogens such as *Porphyromonas gingivalis* reach circulatory systems, causing endothelial dysfunction and promoting breakdown of BBB. Ensuring permeability makes it possible for immune cells and microbial byproducts to enter central nervous system (CNS), exacerbating MS's neuroinflammation and demyelination (5). To our knowledge, this is the first systematic review to synthesize evidence on PD, oral microbiome, and salivary biomarkers in MS, aiming to clarify their potential contribution to neuroinflammation. Hence, this systematic review examines research on epidemiological relationship between adult PD and MS.

## 2 Materials and methods

### 2.1 Research question

The PECO question addressed in the study was: “Is there an increased prevalence or severity of PD in individuals with MS compared to individuals without MS?”

### 2.2 Literature sources

For research, three databases (Scopus, MEDLINE/PubMed, Cochrane Central Register of Controlled Trials—CENTRAL) were examined. Google Scholar and Google were searched manually, and open grey.eu was utilized to assess the Gray literature because the terms “PD” and “MS” were used.

### 2.3 Search strategy

Various electronic databases were used to conduct an electronic literature search, such as PubMed, Scopus, and Cochrane database, by combining the phrases “Periodontal Disease,” “Multiple Sclerosis,” and “Neurodegeneration.” Reference lists of the selected articles were manually screened to discover other relevant articles. Duplicates were removed using Mendeley Reference Manager—Version 2.129.0. The complete search strings for each database are provided in Table 1.

**Table 1:** Search string for the database.

S.no	Database	Search strategy	Articles retrieved
1	Pubmed	(“PDs"[MeSH Terms] OR (“periodontal"[All Fields] AND “diseases"[All Fields]) OR “PDs"[All Fields]) AND (“multiple sclerosis"[MeSH Terms] OR (“multiple"[All Fields] AND “sclerosis"[All Fields]) OR “multiple sclerosis"[All Fields])	68
2	Scopus	TITLE-ABS-KEY (periodontal AND disease AND multiple AND sclerosis) AND (LIMIT-TO (EXACTKEYWORD, “Multiple Sclerosis”) OR LIMIT-TO (EXACTKEYWORD, “PD”)) AND [LIMIT-TO (LANGUAGE, “English”)]	123
3	Cochrane	(“periodontitis”):ti,ab,kw AND (“multiple sclerosis”):ti,ab,kw (Word variations have been searched)	05

### 2.4 Inclusion criteria

Human observational (cohort, case-control, cross-sectional), interventional studies, and clinical studies studying the epidemiological association between PD and MS.
Studies exhibiting microbiological, or immunological associations between PD and MS.

### 2.5 Exclusion criteria

Studies focusing only on PD or MS.
Studies with no clear data on the association between PD and MS,
Studies on languages other than English.
Case reports, editorials, reviews, letters, protocols, book chapters, posters, personal communications, and conference abstracts were excluded.
Animal and *in vitro* studies were excluded

### 2.6 Study selection

Two researchers reviewed the articles retrieved from the extensive database search. Articles were included based on their title, abstract, and study design. Duplicates were removed using Mendeley Reference Manager—Version 2.129.0. The same two reviewers assessed full-text articles independently.

### 2.7 Data extraction

Two independent reviewers screened articles at the title/abstract and full-text stages. Disagreements were resolved by consensus or adjudication from a third reviewer. Although this review was not registered in PROSPERO, PRISMA guidelines were strictly followed to ensure methodological rigor. Newcastle–Ottawa Scale scores ≥7 were considered indicative of high methodological quality.

## 3 Results

### 3.1 Study characteristics

**Figure 1:**
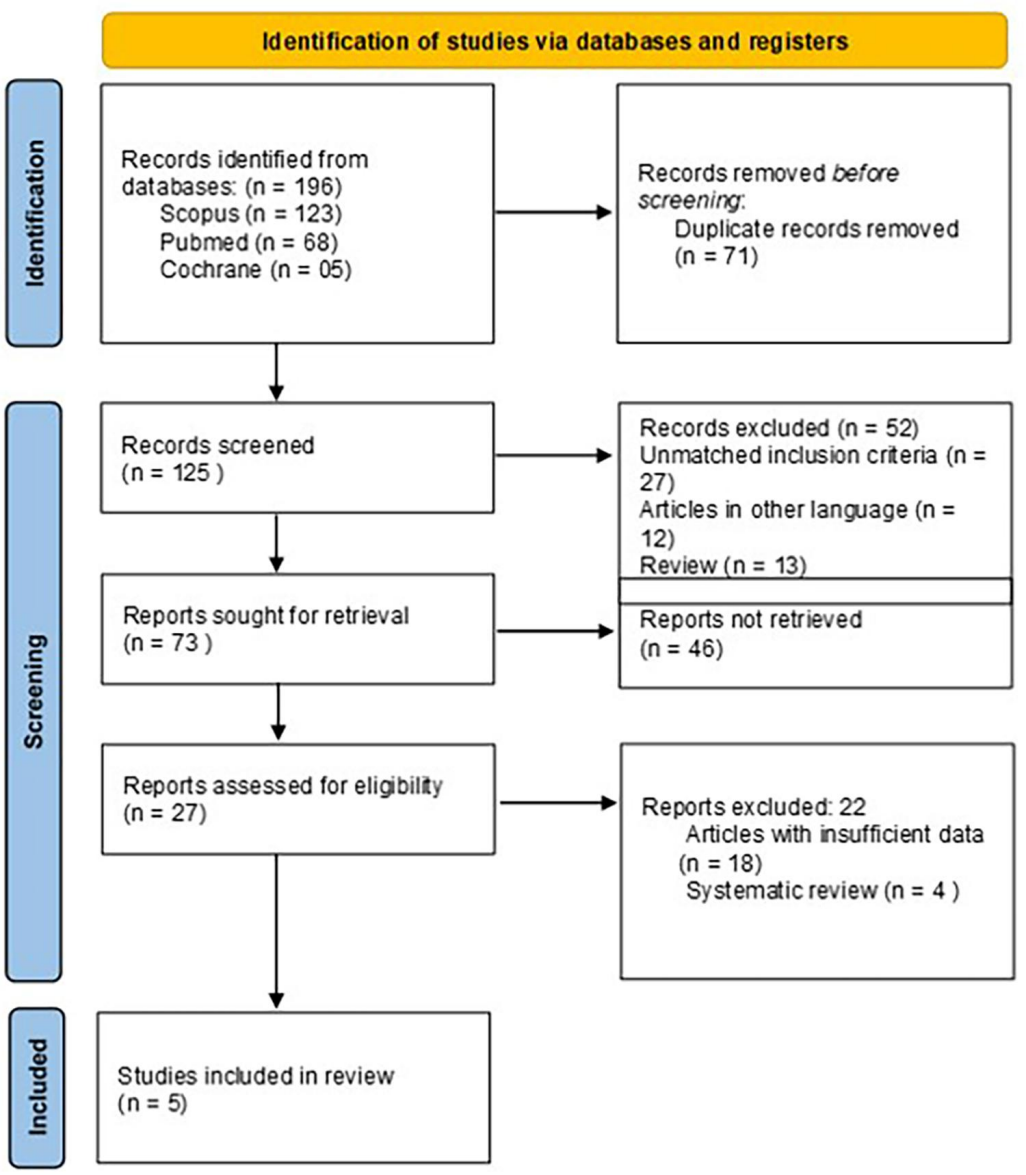
PRISMA flowchart.

Figure 1 illustrates selection process of the studies included. A total of six studies (two cross-sectional studies, three case-control studies, and one pilot case-control study) involving 755 MS patients and 1,774 controls were included in this systematic review. These studies were carried out in five countries namely Taiwan, France, Poland, Turkey, and Germany between 2013 and 2024. Table 2 shows the study characteristics of all the included studies.

**Table 2:** Characteristics of the included studies.

S.no	Study	Location of the study	Study design	Sample size	Exposure	Outcome measures	Data	Results
1.	Sheu et al. (2013) (6)	Taiwan	Case-Control study	Case—316 Control—1,580	1. Periodontal examination—Characteristics of gingiva 2. Probing pocket depth 3. Tooth mobility 4. Radiographs for bone loss assessment	Multiple Sclerosis cases were sourced from administrative databases	Crude OR & Adjusted OR	Subjects exposed to CP had 1.89 times increased chances of being as cases (Multiple Sclerosis) than controls Crude OR = 1.89. After adjusting with potential confounders, the subjects exposed to CP had 1.86 times increased. Both the ORs were found to have significant chances of being cases than controls
2.	Boussamet et al. (2024) (7)	France	Pilot case control study	Case—14 Control—21	Evidence of Periodontal pathogens	McDonald criteria recruited from hospitals	Relative abundance (%)	significant oral dysbiosis was found between Multiple Sclerosis and Hv
3.	Kapel-Reguła et al. (2024) (13)	Poland	Case-Control study	Case—101 Control—51	Approximal Plaque Index (API) and Sulcus Bleeding Index (SBI)	McDonald's criteria	Mean SD, Median IQR	The API was significantly higher, while the SBI was significantly lower in Multiple Sclerosis patients.
4.	Varol et al. (2023) (9)	Turkey	Cross sectional study	Case—92 Control—92	PI, GI, BOP %, PD, CAL, salivary oxidative status, Neutrophil lymphocyte ratio	McDonald's criteria	Mean SD	No significant differences in periodontal parameters between patients with Multiple Sclerosis and Heathy controls. However, Patients with Multiple Sclerosis had significantly higher levels of NLR and TOS than the healthy controls
5.	Auerbacher et al. (2022) (14)	Germany	Case-control study	Case—152 Control—30	Periodontal screening index	Patient records	Mean SD, OR	No significance in periodontal Screening index among Multiple Sclerosis and healthy controls
6.	Hatipoglu et al. (2016) (10)	Turkey	Cross sectional study	Case—80	PI, GI, PD, CAL	McDonald's criteria	Mean SD	Periodontal parameters were found to be significantly higher in H-DS Multiple Sclerosis group than L-DS Multiple Sclerosis

#### 3.1.1 Sample size

Sample sizes varied across studies, with cases ranging from 14 to 316 and controls from 21 to 1,580. The study conducted by Sheu et al. (6) had the highest sample size of 1,896 with 316 cases and 1,580 controls, while Boussamet et al.'s (7) pilot case-control study had the lowest sample size of 35 with 14 cases and 21 controls.

#### 3.1.2 Exposure and periodontal assessment

Periodontal health was assessed using various factors, including: Bleeding on Probing (BOP%), Clinical Attachment Level (CAL), Gingival Index (GI) (8), Plaque Index (PI) (8), Probing Depth (PD) [Varol et al. (9); Hatipoglu et al.] (10). Sulcus Bleeding Index (SBI) (11) and Approximal Plaque Index (API) (12) have been measured by Kapel Reguła et al. (13). Auerbacher et al. (14) received evaluation from Periodontal Screening Index (PSI) (15). Boussamet et al. studied periodontal pathogen relative abundance for oral dysbiosis assessment (7). The heterogeneity of indices limited direct comparability between studies.

#### 3.1.3 Outcome measures

Most studies utilized McDonald's criteria to establish a standard approach for MS diagnosis (16). Patient records served as the basis for identifying MS cases in the study by Auerbacher et al. (14), but Sheu et al. (6) extracted their cases from administrative databases.

#### 3.1.4 Association between periodontal parameters and MS

Sheu et al., (6) proved that subjects exposed to chronic periodontitis had 1.89 times increased chances of having MS than controls (Crude OR = 1.89). After adjusting with potential confounders, Crude OR was found to be 1.86, thus confirming the association between MS and PD.

Hatipoglu et al., (10) found that periodontal parameters such as Periodontal Index (PI), Gingival Index (GI) and Clinical Attachment Level (CAL) were found to be significantly higher in MS patients with high disability than MS patients with low disability.

Kapel-Reguła et al. (13) proved that MS was significantly associated with higher Approximal Plaque Index (API) scores.

Hatipoglu et al. (10) also showed correlation between Expanded Disability Status Scale (EDSS) score and periodontal conditions from which higher EDSS scores was associated with poor periodontal health (*r* = 0.52, *p* < 0.01).

However, Auerbacher et al. (14) showed no significant differences in Periodontal Screening index among MS and healthy controls.

Similarly, no significant differences were observed in periodontal parameters between patients with MS and Healthy controls in the study conducted by Varol et al. (9).

#### 3.1.5 Association between periodontal biomarkers (oral microbiota) and MS

Boussamet et al. explored the metabolite signature of oral microbiota in MS patients, identifying significant microbial composition shifts using 16S rRNA sequencing. They established that the degree of oral microbiota decreased, commensal bacteria were reduced, while pathogenic species increased (*p* < 0.01) (7). Two studies investigated salivary biomarkers as potential disease progression indicators in individuals diagnosed with MS. Kapel-Reguła et al. examined the level of cortisol and reported that level of stress biomarker in patients with MS has been significantly higher than control group (7.5 ± 2.1 vs. 4.2 ± 1.8 ng/mL, t = 6.3, *p* < 0.01) and associated with poor oral health and missing teeth (13). For instance, Varol et al. established that there was a higher level of oxidative stress in saliva of the patients with MS in terms of malondialdehyde (MDA) levels (3.2 ± 0.8 nmol/mL vs. 1.5 ± 0.5 nmol/mL, *p* < 0.05), indicating immune system and inflammation influencing oral health (9). The reviewed studies confirm that MS patients experience significant oral health challenges, having high prevalence of PD, changes in oral microbiota, increased levels of stress and oxidative damage biomarkers in saliva, and reduced quality of life due to oral diseases.

### 3.2 Risk of bias assessment

**Figure 2:**
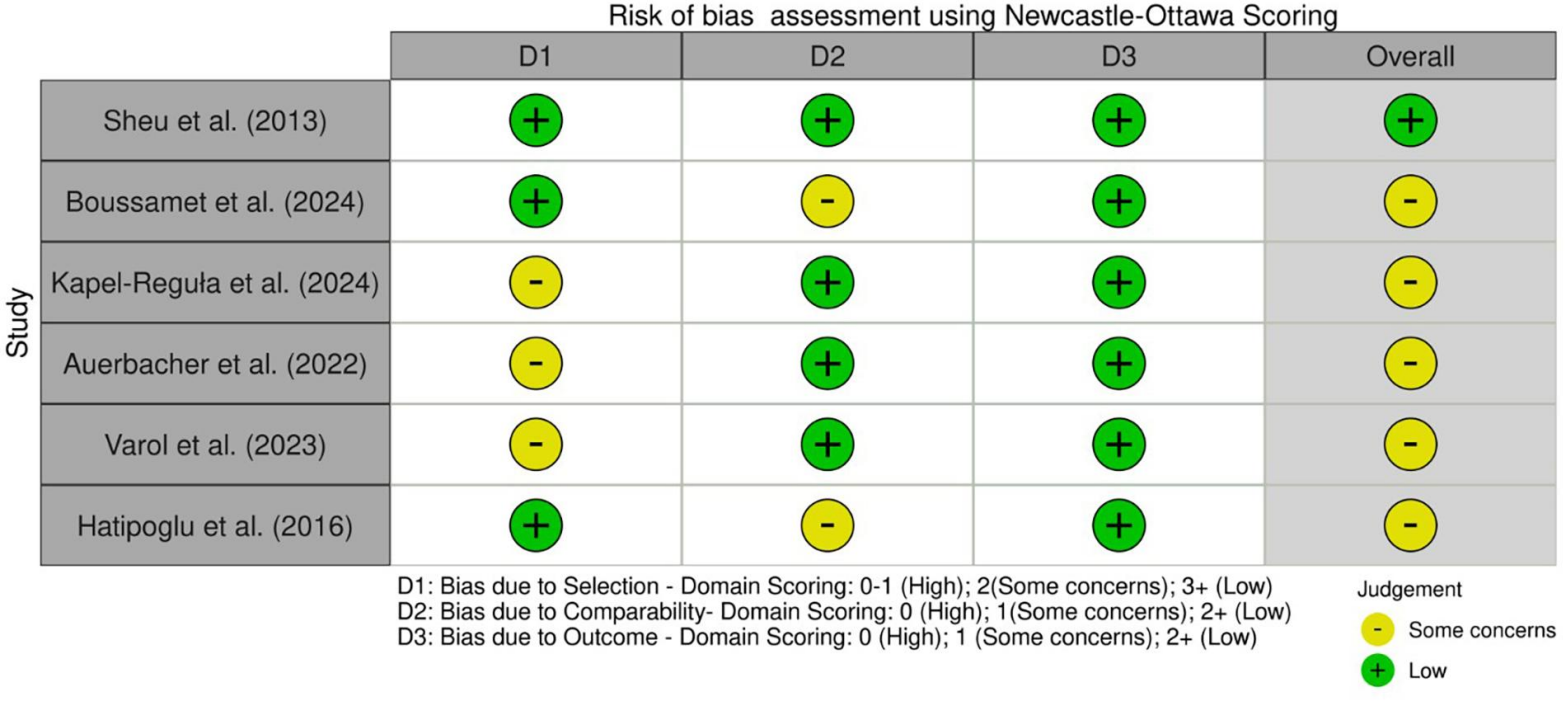
Risk of bias assessment using Newcastle-Ottawa scoring.

The included studies were evaluated using the Newcastle-Ottawa Scale (NOS), which assesses the quality of non-randomized studies across three domains: Selection (4 points), Comparability (2 points), and Exposure/Outcome (3 points), with a maximum score of 9. Five studies (7, 9, 10, 13, 14) scored 6–8 points. One study (6) scored 9 points. In overall, the study conducted by Sheu et al. (6) alone had low risk of bias whereas all other studies had some concerns. No studies were rated as having a high risk of bias. (Figure 2) Overall, the evidence base is of moderate quality, with most studies showing some concerns regarding comparability of cases and controls.

## 4 Discussion

This review identified six observational studies suggesting positive association between MS and PD due to the findings of altered oral microbiota, higher scores of indices related to periodontitis, and elevated salivary inflammatory biomarkers among MS patients compared to healthy controls. The study results suggest that there is a close and complicated connection between PD and MS, proving role of inflammation and dysbiosis in development of MS. This systematic review outcome showed a complex interaction between MS and PD, with the focus on the impact of periodontal inflammation and oral dysbiosis on neuroinflammation in MS patients.

PD is an inflammatory condition that weakens the teeth and their supporting tissues, culminating in loss of dentition. The disease is therefore a result of a multifactorial etiology that influences changes in the pathogenesis. Some of the factors include: poor oral hygiene, stress, immune-compromised conditions, diabetes, hormonal disturbances, and other environmental factors that contribute to the worsening of PD (17). Many studies examined possible consequences of the inflammatory PD on several organ systems. Moreover, certain systemic diseases can affect host's immune response, increasing rate of periodontal tissue breakdown compared to that of regular host (18–20). Central nervous system is affected by chronic, and autoimmune disease known as MS. This immune-mediated inflammatory disease that affects myelinated axons, causing significant disability (21).

In this review, several studies showed a significant association between MS and PD. For example, Hatipoglu et al. found that PD is related to more severe disability scores among patients with MS, while Sheu et al. noted that chronic periodontitis is more common in patients with MS (6, 10). Moreover, Boussamet et al. reported significant differences in oral microbiota in patients with MS, indicating that the patients had dysbiosis and the changes affected the whole body (7, 22). These results are consistent with growing data linking neurodegenerative disorders to systemic inflammation and microbial dysbiosis (22, 23). Despite most of the studies showing positive link between periodontal health and MS, few studies had contrasting results. Auerbacher et al. in their study didn't find any significant associations between Periodontal Screening Index parameters and neurodegenerative disorder (14). Moreover, Varol Ç et al. (9), also found no significant associations between healthy controls and MS patients for periodontal parameters. It is advocated that oral health problems in MS is multi-faceted (9). While few studies have precisely mentioned the confounders influencing the cause of MS, few studies have failed to appropriately adjust to all the confounders such as smoking, oral hygiene and etc. The contrasting results could be attributed to these confounding factors which are independently associated with both periodontitis and MS. Also, the case definitions, indices and assessment methods used by all these studies were highly varied. The varying results could also be due to these methodological heterogeneities.

Exact molecular pathways between PD and MS remain unidentified despite possible biological pathways existing between these diseases. Presence of elevated cytokines, including interleukin-1 beta(IL-1β), interleukin-6(IL-6), and tumour necrosis factor-alpha(TNF-α), indicates that PD leads to systemic inflammation (24, 25). Mediators possess destructive effect on blood-brain barrier (BBB), thus enabling it to become more penetrable to immune cells and microbial material that enter the central nervous system (CNS) (26–28). During this process, inflammatory response intensifies, which worsens and aggravates two dominant characteristics of MS: neuroinflammation and demyelination. During periodontal infections, the virulence factors released by *Porphyromonas gingivalis* and *Treponema denticola* produce lipopolysaccharides (LPS) that drive additional immune responses, possibly related to CNS inflammation (29, 30). This supports the concept of an oral–immune–neuro axis, paralleling evidence from gut microbiome studies in MS.

The present review on salivary biomarkers strengthens the existing relationship between PD and MS. The research by Kapel-Reguła et al. suggests that MS patients showed elevated cortisol levels in their saliva, possibly indicating stress-based immunological changes due to poor dental health (13). The research by Varol et al. revealed that MS patients showed elevated levels of malondialdehyde (MDA) in their saliva, which could point to increased inflammation affecting periodontal tissues and brain tissues (9). The study findings confirm the concept that both MS-related immunological irregularities and systemic oxidative stress contribute to the worsening of PD as well as worsening MS symptoms.

The risk of bias assessment suggests a moderate quality of evidence of all the included studies. All the six studies either included standardized assessment criteria or universally accepted indices to measure exposure and outcomes, thus limiting the chances of bias due to outcome/exposure assessment. There were few concerns in the way confounders were handled in few studies; however they didn't affect the comparability of cases and controls much. Similarly, with respect to selection bias, none of the studies showed high risk of bias. Hence, in overall, the present review suggests a moderate quality of evidence for association between periodontitis and MS. However, considering variations in the case definitions, diagnostic tools and summary measures used among the studies, a quantitative meta-analysis was not performed.

This systematic review has few limitations. Only six studies were identified in this review, thus limiting the level of evidence pertaining to association of PD and MS. In the present review, articles published only in English language (or English translation available) were included, thus limiting the number of articles included in the review. Secondly, due to reporting and methodological differences present among studies included, quantitative synthesis was not feasible. The methodological limitations such as bias related to search strategy, selection, data extraction and risk assessment were avoided in best possible ways. However, limitations related to evidence existed. Despite not having any major issues with respect to quality of the studies, nothing much can be commented on the strength of evidence or Hill's criteria of causal relationship as all the included studies were observational studies (mostly case-control and cross-sectional). The heterogeneity of the studies with respect to periodontal assessment, sample size and outcome measures could also deter the quality of evidence obtained. Also several studies failed to adjust for key confounders (such as smoking, immune-modulatory therapies and oral hygiene habits) thus leading to compromised validity of reported associations. The results were not expressed in terms of relative risk/odds ratio in most of the studies. This curbs the understanding of strength of association between PD and MS. Moreover, considering the study designs of the articles included in the study, the consistency of the results regarding the association between PD and MS is questionable, particularly in cohort/longitudinal studies. Hence, longitudinal research with standardized research methods needs to be performed to establish a causal link between PD and MS. Also, absence of experimental/interventional studies in the review highlights the importance of interventional studies on MS patients to improve periodontal health.

However, within the limitations of the review, it could be accepted that PD is associated with MS. Hence, periodontal health maintenance emerges as a vital element of MS treatment. Routine dental examinations, professional dental scaling, and proper oral hygiene counseling present the potential to decrease systemic inflammation and boost overall health outcomes for MS patients. Research must analyze how scaling and root planing alongside antibiotic therapy affect the progression of MS symptoms.

## 5 Clinical implications

Given the bidirectional link between systemic inflammation and oral health, routine periodontal screening and preventive care should be integrated into the multidisciplinary management of MS patients. Whether periodontal therapy can reduce neuroinflammation or modify disease progression warrants testing in prospective intervention studies.

## 6 Conclusion

This systematic review demonstrates that periodontal health is vital for MS patients as periodontal inflammation and oral dysbiosis seem to promote both systemic immune activation and neuroinflammatory processes. Future longitudinal and interventional studies with standardized periodontal criteria are needed to establish causality. Until then, maintaining periodontal health remains a pragmatic strategy to potentially mitigate systemic inflammation in MS.

## Data Availability

The original contributions presented in the study are included in the article/Supplementary Material, further inquiries can be directed to the corresponding author.
